# MicroRNA Interference in Hepatic Host-Pathogen Interactions

**DOI:** 10.3390/ijms22073554

**Published:** 2021-03-30

**Authors:** Asahiro Morishita, Kyoko Oura, Tomoko Tadokoro, Koji Fujita, Joji Tani, Tsutomu Masaki

**Affiliations:** Department of Gastroenterology and Neurology, Kagawa University School of Medicine, Kagawa 761-0793, Japan; kyoko_oura@med.kagawa-u.ac.jp (K.O.); t-nishioka@med.kagawa-u.ac.jp (T.T.); 92m7v9@med.kagawa-u.ac.jp (K.F.); georget@med.kagawa-u.ac.jp (J.T.); tmasaki@med.kagawa-u.ac.jp (T.M.)

**Keywords:** diagnostic biomarker, liver immunity, immune cells, host–pathogen interaction, microRNA, RNA interference, epigenetic changes, pathogen

## Abstract

The liver is well recognized as a non-immunological visceral organ that is involved in various metabolic activities, nutrient storage, and detoxification. Recently, many studies have demonstrated that resident immune cells in the liver drive various immunological reactions by means of several molecular modulators. Understanding the mechanistic details of interactions between hepatic host immune cells, including Kupffer cells and lymphocytes, and various hepatic pathogens, especially viruses, bacteria, and parasites, is necessary. MicroRNAs (miRNAs), over 2600 of which have been discovered, are small, endogenous, interfering, noncoding RNAs that are predicted to regulate more than 15,000 genes by degrading specific messenger RNAs. Several recent studies have demonstrated that some miRNAs are associated with the immune response to pathogens in the liver. However, the details of the underlying mechanisms of miRNA interference in hepatic host–pathogen interactions still remain elusive. In this review, we summarize the relationship between the immunological interactions of various pathogens and hepatic resident immune cells, as well as the role of miRNAs in the maintenance of liver immunity against pathogens.

## 1. Introduction

### 1.1. Inflammatory Processes and Liver Homeostasis

The liver used to be recognized as a non-immunological viscera that orchestrates metabolic activities, nutrient storage, and detoxification. The results of many recent studies have demonstrated that resident immune cells are responsible for various immunological reactions in the liver, leading to the production of cytokines and chemokines [1,2,3]. As an anatomical structure, the liver is constantly exposed to many nutrients following their absorption in the gut, as well as commensal bacterial products that cause immune responses [2]. The liver immune system neutralizes toxic bacterial products and protects the body [2]. Constant exposure to bacterial products results in the modulation of inflammation [4]. The inflammatory processes are strictly regulated in the normal liver, but additional activity, which is required to remove hepatotropic pathogens (including malignant cells or toxic products), disturbs the hepatic immune system [3]. Dysfunction of the hepatic immune system causes chronic infection, autoimmunity, or hepatocarcinogenesis. This leads to chronic inflammation and disruption of tissue homeostasis, resulting in hepatic fibrosis, cirrhosis, and hepatocellular carcinoma (HCC) [4]. In addition, homeostatic inflammatory processes (which are tightly regulated) modulate hemodynamic changes, capillary permeability, inflammatory cell migration into tissues, and the secretion of inflammatory cytokines, which resolves inflammation and promotes tissue regeneration. This inflammatory process is essential for maintaining liver homeostasis. However, the mechanism(s) underlying its dysregulation remains elusive [4].

### 1.2. Organization of the Liver Inflammatory Response

The liver plays a critical role as a pivotal buffer between the contents absorbed through the digestive system and systemic circulation. Most of the blood supply to the liver flows through the portal vein [2]. The blood supply in the liver is abundant with harmless nutrition-rich antigens, as well as bacterial antigens from the gut microflora [2]. Immunosurveillance is one of the most important tasks of the liver for controlling pathogenic infections [3]. Venous blood from the gut and oxygen-rich blood from the hepatic artery are mixed in the sinusoids and drain to the central veins in the liver [2]. Liver sinusoidal endothelial cells (LSECs) are located in the sinusoids, which enable the rapid exchange of molecules between the blood and hepatocytes [2]. LSECs also enable the degradation of harmful immunogens. Kupffer cells (KCs) and hepatocytes express pattern-recognition receptors (PRRs), which bind to microbial-associated molecular patterns (MAMPs) [5,6] and damage-associated molecular patterns (DAMPs) [7], and subsequently phagocytose and degrade MAMPs and DAMPs without inducing inflammatory mediators, which results in detoxification of the blood. This function of the liver defends the body from excessive immune activation. In addition, sinusoidal blood flow with low pressure, fenestrated endothelial cells, and the absence of a basement membrane [8] facilitate interactions between resident immune cells and non-hematopoietic hepatic cells. These resident immune cells include professional antigen-presenting cells, as well as innate and adaptive lymphoid cells [9,10,11]. Although several resident immune cells in the liver, such as KCs, have been well analyzed, the full spectrum of hepatic resident immune cells remains unclear. Moreover, the interactions between these resident immune cells and various liver pathogens are unknown.

### 1.3. MicroRNAs (miRNAs, miRs)

Small non-coding RNAs include miRNAs, which are endogenous, interfering RNAs, as well as small interfering RNA, and P-element-induced wimpy testis-interacting RNA. Over 2600 miRNAs have been discovered and are predicted to modulate more than 15,000 genes [12] via suppression of translation by binding to the 3′-untranslated region of target messenger RNAs (mRNAs). Remarkably, one miRNA can regulate more than 200 mRNAs [13,14].

The biogenesis of miRNAs involves several steps, including transcription, cleavage, export, and interactions with mRNAs [15]. MicroRNA genes are initially transcribed by RNA polymerase II and processed via the canonical pathway or the mirtrons pathway as primary miRNA transcripts (pri-miRNAs) that consist of one or more hairpin structures [16]. Pri-miRNAs are capped on the 5′ end and polyadenylated on the 3′ end. Subsequently, they are cleaved into ~70-nucleotide (nt) heparin-structured precursor miRNAs (pre-miRNAs) with a 5′ phosphate and a 3′ two-nt overhang by a multiprotein complex comprising Drosha, which is an RNase III enzyme, and DGCR8 or Pasha, which are double-stranded RNA-binding domain proteins (dsRBD) [17]. Exportin-5 then translocates pre-miRNAs from the nucleus to the cytoplasm via a Ran-GTP-dependent mechanism [18]. These translocated pre-miRNAs are cleaved by Dicer, which is a second RNase III endonuclease, and TRBP/PACT, which is a dsRBD [19]. Finally, one of the strands of the pre-miRNA interacts with an argonaute (AGO) protein, resulting in the cleavage of the pre-miRNA in the RNA-induced silencing complex (RISC) [20].

Several reports have demonstrated that a number of miRNAs are involved in the immune response to pathogens in various liver diseases. However, the underlying mechanistic details remain unclear. Further analyses are required to elucidate the relationship between the immunological interaction of the pathogens by hepatic resident immune cells and miRNA interference in the liver.

In this review, we summarize the relationships between immune responses and miRNA interference for various pathogens in the liver.

## 2. Relationships between miRNAs and Immune Responses in the Liver

### 2.1. Immune Responses in the Liver

The liver has a unique anatomy in that it receives blood flow from both the hepatic artery and the portal vein and is constantly presented with antigens of gastrointestinal origin, such as food-associated antigens and pathogen-associated molecular patterns (PAMPs) via the portal vein [21]. The liver is generally known as an immune-permissive organ, and various immunocompetent cells in the liver, such as dendritic cells, KCs, natural killer (NK) cells, natural killer T cells, and regulatory T cells, help maintain a balance between immune responses and tolerance to foreign antigens [21,22]. This innate immune system recognizes PAMPs, which are common molecular structures produced by bacteria and other organisms [23]. Toll-like receptors (TLRs) have been identified as receptors that recognize pathogens [24]. Immune cells in the liver sinusoids express PRRs, such as TLRs, which recognize different components of pathogens. Although many studies have contributed significantly to our understanding, signaling through a series of PRRs (such as TLRs) is still not fully understood [25]. Recently, miRNAs have attracted attention as regulators of pathogenic infection [26]. The roles of miRNAs as regulators of cellular functions have also attracted attention, and miRNAs have been demonstrated as important in various physiological and pathological conditions [27].

In addition to epigenetic interference by miRNA, classical epigenetic mechanisms, including DNA methylation and histone acetylation, which affect the host–pathogen immunity without changing nucleotide sequences, are known [28]. In fact, some parasites can influence immune responses through histone modifications [29]. Epigenetic changes, other than miRNA interference, have also been recognized as important mechanisms causing allergic reactions in response to pathogens. Further studies may reveal more accurate mechanisms of immunological interactions between the pathogen and host.

### 2.2. MicroRNAs in the Immune System

Innate immunity reactions are centered on neutrophils, macrophages, and dendritic cells. MicroR-233 suppresses excessive differentiation and proliferation by negatively regulating neutrophil differentiation [30]. MicroR-155 regulates interleukin (IL)-1 signaling by controlling the expression of TAK1 binding protein 2 (TAB2), a protein associated with the Toll-like receptor–IL-1 pathway in dendritic cells [31]. In addition to innate immunity, miRNAs are also involved in various acquired immune reactions such as T cell or B cell differentiation, antigen presentation, immunoglobulin class switching, cytokine production, and T cell receptor signaling [32]. Phosphatases such as mSHP2, PTPN22, DUSP5, and DUSP6 act as negative regulators of T cell activation, and miR-181a regulates T cell reactivity by lowering their expression levels [33,34]. MicroR-155 also affects acquired immunity and maintains the homeostasis of regulatory T cells by regulating the expression of suppressor of cytokine signaling (SOCS) 1, which is a negative regulator of IL-2 receptor signaling [33,35]. MicroRNAs influence both innate and adaptive immune responses and, thus, impact the outcomes of liver diseases.

### 2.3. MicroRNAs and Immunity in the Liver

MicroR-122 is the most plentiful miRNA, specifically expressed in hepatocytes, and accounts for approximately 70% of all miRNAs in the liver [36,37]. It targets three receptor-type tyrosine kinases—namely, MERTK, FGFR1, and IGF1R, which directly promote STAT3 phosphorylation and play a pivotal role in regulating the innate immunity of hepatocytes [38]. MicroR-155, a common target of many inflammatory mediators, also plays a critical role in the immune response of the liver [39]. It participates in both innate and adaptive immune responses. The expression levels of miR-155 are changed in both liver tissue and circulating inflammatory cells by liver injury; therefore, maintaining miR-155 expression in inflammatory cells is important for regulating liver injury.

## 3. MicroRNAs and Immune Responses against Viral Infection in the Liver

**Figure 1:**
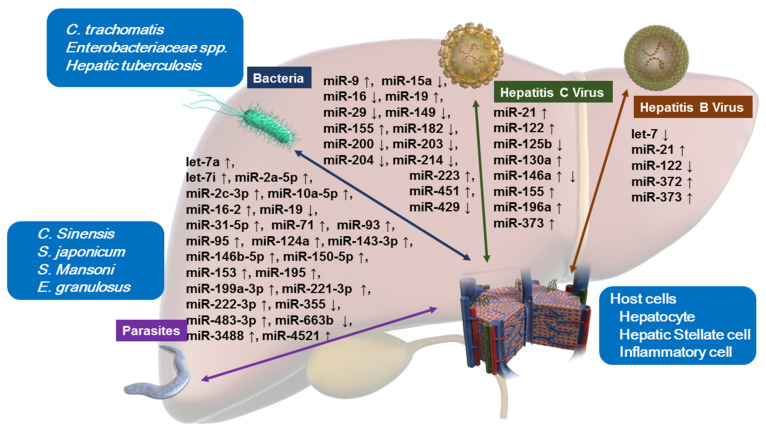
Altered miRNA expressions upon host–pathogen interactions in hepatocytes, hepatic stellate cells, and inflammatory cells. Bacteria: *Chlamydia trachomatis*, *Enterobacteriaceae* spp., *Mycobacterium tuberculosis* (hepatic tuberculosis). Parasites: *Clonorchis sinensis*, *Schistosoma japonicum*, *Schistosoma mansoni*, *Echinococcus granulosus*.

Here, we review the miRNA function related to the interactions between host cells and various pathogens (Figure 1, Table 1).

### 3.1. Interaction between Host miRNAs and Hepatitis Viruses

Chronic inflammation in the liver induces pro-inflammatory cytokines, including tumor necrosis factor-alpha (TNFα) and Interleukin-6 (IL-6), through the activation of KCs and liver-derived macrophages. Pro-inflammatory cytokines evoke both pro-apoptotic and anti-apoptotic effects in liver tissues with injury [71,72].

In a pro-apoptotic response, inflammation/necrosis triggers TLR signaling, activating KCs to synthesize pro-inflammatory cytokines, resulting in NK cell recruitment, which promotes the expression of TNF-related apoptosis-inducing ligand [73,74,75]. In contrast, an anti-apoptotic response is induced by the activation of the canonical nuclear factor-κB (NF-κB) inflammation pathway [76]. Injury stimulates hepatic stellate cells and KCs to produce chemokines such as TNFα and IL-6, which stimulate TLR signaling pathway in hepatocytes. The activation of TLR signaling then phosphorylates the IαKα–p65:p50 complex in the cytoplasm through the inhibition of kappa kinase complex, resulting in translocation of p65:p50 in the nucleus. This accumulation of p65:p50 in the nucleus progresses the anti-apoptotic response [71,77,78].

In the context of chronic liver inflammation, miRNAs in host cells can be categorized as proviral or antiviral based on their functions when a virus enters the liver. Several host miRNAs perform antiviral actions by regulating viral replication, inhibiting proviral proteins, or inducing the virus to transfer to a latent phase [79,80,81,82]. Some other miRNAs exert a proviral function, assisting replication and infection of the virus [83,84,85], suppressing antiviral factors, and helping the virus evade the immune response in host cells [85,86]. 

Furthermore, viral infection suppresses the expression of miRNAs that modulate immune responses, cell proliferation, and DNA repair [37,87].

### 3.2. MicroRNA Signaling in Hepatitis B Virus (HBV) Infection

MicroR-122, which is a liver-specific miRNA, plays a pivotal role in lipid metabolism, tumor suppression, and liver homeostasis [88]. HBV replication is suppressed by miR-122, which enhances p53 activity by reducing cyclin G1 expression [89,90]. Moreover, HBV replication is inhibited by regulation of the interferon (IFN)-signaling pathway. IFN circuitry can be hindered via SOCS overexpression, which occurs as a result of miR-122 downregulation [43].

In addition to miR-122, upregulation of miR-372 and miR-373 promotes HBV protein expression and replication, thus favoring viral progression by targeting the nuclear factor I/B-dependent pathway [44].

Members of the let-7 family of miRNAs are reduced in chronic hepatitis B by the HBx protein. Toll-like receptor 4 signaling is inhibited by let-7 family and loss of the function of this family in the IL-1 receptor associated kinase (IRAK1)–tumor necrosis factor receptor-associated factor 6 (TRAF6)–NF-κB pathway induces the nuclear translocation of p65 and p50 [91] and induces signal transducer and activator of transcription 3 (STAT3) expression, resulting in cell proliferation and survival [40,78]. Alternatively, HBx downregulates let-7, which can also stimulate STAT3 signaling by controlling the failure of the production of inflammatory cytokines [92]. HBx-induced miR-21 overexpression by IL-6-induced STAT3 signaling blockades tumor suppressors such as phosphatase and tensin homologue and programmed cell death homologue 4 in the various stages of inflammation, fibrosis and HBV-induced HCC [41,42].

### 3.3. MicroRNA Signaling in Hepatitis C Virus (HCV) Infection

MicroR-155 is involved in the activation of many inflammatory mediators and enhances both autoimmune inflammation by increasing inflammatory T cells [39] and immune responses during HCV infection [53]. HCV induces miR-155 expression in vitro and in vivo. In fact, miR-155 is more plentiful in liver tissue and serum samples from HCV-infected patients than uninfected control subjects [52,54]. The expression of miR-155 significantly decreased in patients who cleared HCV after treatment, supporting the concept that the enhancement of miR-155 assists HCV infection [54]. MicroR-155 modulates the innate immune response by influencing the expression of IFN-regulated genes in NK cells during chronic HCV infection [93] and controlling the immune response to HCV in the liver [53].

HCV enhances the expression of miR-21, which plays an important role in regulating the targeting elements of the TLR signaling pathway, such as IRAK1 and IRAK4, TRAF6, and myeloid differentiation primary response 88 (MyD88) protein in liver tissues, inducing the suppression of type-I IFN to evade the immune response [45].

MicroR-122, which is called a liver-specific miRNA, can be enhanced by HCV infection and is a predictive biomarker of a poor response to IFN therapy [47]. Its upregulation tends to induce SOCS3 expression, thereby reducing STAT3 activation and the promotion of antiviral genes through the interferon-stimulated gene factor (ISGF) 3 [46].

The relationship between miR-146a expression levels and HCV infection may be complex. MicroR-146a is naturally plentiful in peripheral blood mononuclear cells (PBMCs) and modulates host immune responses [51]. MicroR-146 was previously shown to be enhanced in HCV-infected hepatocytes and liver tissues [51]. However, diminished expression of miR-146a has also been detected in PBMCs in patients with chronic hepatitis C. This can partially be attributed to differences in the HCV genotype.

MicroR-130a appears to serve a bivalent role in HCV infection. Its expression in liver tissues is much higher in patients with HCV infection than in normal subjects. Knocking down miR-130a expression suppresses HCV RNA replication in hepatocytes by promoting interferon-induced transmembrane protein 1 (IFITM1) [50]. HCV may enact a strategy to maintain persistent infection by upregulating miR-130a and downregulating IFITM1, and thereby benefiting from the subsequent innate immune response. In contrast, miR130a overexpression also suppresses HCV RNA replication with both the Con1b replicon and the JFH1-based cell culture system [49]. Finally, miR-130a downregulates miR-122 but upregulates proteins that coordinate innate host immune responses, including type I IFN (IFNα/IFNβ), interferon-stimulated gene 15 (ISG15), ubiquitin specific peptidase 18 (USP18), and myxovirus resistance protein 1 (MxA). Therefore, these data suggest that miR-130a has dual functions in HCV replication and the subsequent host immune response.

MicroR-196a inhibits Bach1 expression and induces heme oxygenase 1 (HMOX1) expression, resulting in inhibited HCV gene expression and replication [55]. Circulating miR-196a expression levels are significantly diminished in chronic hepatitis C patients, in spite of the amount of HCV or alanine aminotransferase levels, indicating that miR-196a release decreases in HCV-infected hepatocytes. Therefore, miR-196a can potentially be used to predict chronic HCV infection in its early stages [94]. Furthermore, miR-196a expression can be enhanced in hepatoma cells after exposure to IFNβ [95]. In addition, miR-373 improves IFN resistance by impairing the Janus kinases (JAK)/STAT signaling pathway [56].

MicroR-125b levels inversely correlate with cytokine expression after challenge with the HCV core protein [48]. Under stimulation of the HCV core protein, miR-125b expression was diminished and cytokine expression was increased in THP-1 cells which are human acute monocytic leukemia cells. Enhanced miR-125b expression can inhibit cytokine expression caused by the HCV core protein through the suppression of NF-κB p65, extracellular signal-regulated kinase, and p38 phosphorylation. In summary, miR-125b may inhibit HCV-induced immune responses by suppressing TLR2 and MyD88 signaling in monocytes [48].

## 4. MicroRNAs and Immune Response against Bacterial Infection in the Liver

Monocytes, macrophages, and dendritic cells, which control infection, can sense and eliminate bacteria that invade the body. This innate immune system recognizes PAMPs, which are common molecular structures of bacteria, and activates intracellular signal-transduction systems [96]. Several studies have identified the signals mediated by receptors that recognize bacteria, including those generated by TLRs, but the associated mechanisms have not been completely elucidated [97]. However, miRNAs have been shown to play pivotal roles in controlling bacterial infections. When studying the innate immune mechanisms occurring in the liver by examining miRNAs involved in bacterial infections, it is possible to understand the mechanisms of miRNA transmission between hepatocytes in greater detail.

### 4.1. Bacterial Liver Abscesses

A liver abscess is defined as a pus-filled mass in the liver that results from the invasion and proliferation of bacteria and protozoa and is caused by liver injury or an intra-abdominal infection disseminated from the portal circulation [98]. The majority of these abscesses are categorized as bacterial or amoebic liver abscesses, depending on the cause of infection. Additionally, depending on the infection route involved, liver abscesses are classified as transbiliary, portal vein, transarterial, traumatic, or idiopathic abscesses, among which transbiliary abscess is the most common. With a bacterial liver abscess, cholangitis caused by bile duct obstruction, which may be caused due to common bile duct stones or malignant tumors of the pancreatobiliary system, spreads in a transbiliary manner into the liver to form an abscess. With an idiopathic liver abscess, the important prerequisite is a state in which the host’s immune system is weakened, such as with diabetes, liver cirrhosis, or cancer [99,100]. Gram-negative bacilli such as *Klebsiella* spp. and *Escherichia coli* are common causative bacteria of liver abscesses; *Streptococcus* and *Staphylococcus* spp. can also be causative bacteria for liver abscesses.

*Klebsiella pneumoniae* is an opportunistic pathogen that causes nosocomial and community-acquired infections and releases outer membrane vesicles (OMVs). OMVs play a role as vehicles for transporting virulence factors to host cells. It was recently reported that four miRNAs, including miR-21, miR-25, miR-223, and let-7g, were dysregulated in human bronchial epithelial cells after an interaction with OMVs produced by *Klebsiella* infection [101]. These miRNAs regulate host immune responses involving TLR4 (miR-21), cytokine production (miR-25), NF-κB (miR-223), and IL-6 (let-7g). Data from another study showed that miR-23a and miR-155 were downregulated in *Klebsiella*-infected pulmonary epithelial cells and that these miRNAs regulated integrin α5β1 function and *Klebsiella* adhesion [65]. Thus, although miRNA regulation of *Klebsiella* infection in the respiratory organs has been reported, little is known regarding this phenomenon in the liver.

### 4.2. Spontaneous Bacterial Peritonitis (SBP)

SBP is a common complication in patients with decompensated cirrhosis and is defined as the presence of infectious lesions without a defined bacterial source for the same in the abdominal cavity, such as in the case of gastrointestinal perforation [102]. SBP occurs in 7–20% of cases of decompensated cirrhosis with ascites and is particularly frequent in alcoholic liver disorder because of impaired reticuloendothelial function [103]. SBP development is associated with decreased bacterial translocation, intestinal peristalsis, and reticuloendothelial function due to the portal-systemic shunt. During liver cirrhosis, portal hypertension and undernutrition cause atrophy and edema of the intestinal mucosa, and intestinal bacteria can easily move into the portal vein or abdominal cavity. These bacteria are usually eliminated by the bactericidal actions of neutrophils and KC phagocytosis in the liver sinusoids. However, in patients with liver cirrhosis, reticuloendothelial function declines, and the biological filter of KCs does not function sufficiently, resulting in idiopathic bloodstream and lymphatic SBP. Gram-negative bacteria, especially *Enterobacteriaceae* spp., are the most frequent etiological agents of SBP, although Gram-positive bacteria appear to be increasingly implicated in SBP [104,105].

Data from a previous study showed that serum miR-122 levels were diminished in patients with hepatic decompensation, compared with patients with compensated liver disease, and patients with SBP can exhibit significantly lower miR-122 levels than those without complications [59]. Data from another study, using blood samples from cirrhosis patients with ascites, showed that miR-155 was a good diagnostic marker for SBP, and a greater efficiency in diagnosis was achieved by detecting both serum CD64 and calprotectin levels [62]. Furthermore, in studies examining ascites miRNA levels in patients with cirrhosis, miR-155 [61] and miR-223 [106] were elevated in patients with SBP, indicating that these miRNAs might be involved in the local immune response during SBP.

### 4.3. Fitz–Hugh–Curtis Syndrome

Fitz–Hugh–Curtis syndrome (also known as perihepatitis) is a chronic symptom of pelvic inflammatory disease that is characterized by inflammation of the hepatic capsule with formation of adhesions and right upper abdominal pain [107]. Pelvic peritonitis, which is derived from cervical inflammation that develops as a sexually transmitted disease, spreads in the abdominal cavity and leads to perihepatitis. *Neisseria gonorrhoeae* is a rare causative organism, and most recent cases of Fitz–Hugh–Curtis syndrome are due to *Chlamydia trachomatis* [108].

A study conducted using a mouse model of *C. trachomatis* demonstrated that miRNAs possibly control the epithelial–mesenchymal transition, fibrosis, and tumorigenesis. *C. trachomatis* infection affected miRNAs that play critical roles in epithelial function, viability, and maintenance, as well as cell cycle regulation (miR-15a, miR-16, miR-29, miR-203, miR-204, miR-200, and miR-429), were downregulated, whereas miRNAs involved in the NF-κB pathway, tumorigenesis, and cell integrity (miR-9, miR-19, and miR-451) were upregulated [57,58]. Furthermore, depletion of miR-182 increased the occurrence of pathologies in *C. trachomatis*-infected mice [63]. Data from another in vivo study demonstrated that miR-214 was significantly downregulated following infection with *Chlamydia muridarum*, another causative agent of genital infection, compared with mock-infected mice, resulting in the upregulation of intracellular adhesion molecule 1 [64].

### 4.4. Hepatic Tuberculosis (TB)

*Mycobacterium tuberculosis* usually infects the lungs but can infect almost any organ, such as the lymph nodes, colon, liver, and spine, causing an extrapulmonary infection. Hepatic TB infection is an extrapulmonary manifestation of active TB infection [109]. Hepatic TB is categorized as miliary or sporadic. Miliary TB develops as a liver lesion with systemic hematogenous dissemination, whereas sporadic TB forms a lesion only in the liver.

Several miRNAs may be involved in the pathogenesis of extrapulmonary TB (EPTB) and pulmonary TB (PTB). In a study designed to investigate the associations between EPTB and single nucleotide polymorphisms (SNPs) in precursor miRNAs, it was found that patients with TB had significantly lower miR-149 levels than healthy control subjects (HCSs). In addition, the C allele of SNP rs2292832 in miR-149 was involved in a higher risk of EPTB compared with that of HCSs, while the A allele of SNP rs71428439 in miR-149 was protective for EPTB and PTB [60].

## 5. MicroRNAs and Immune Responses against Parasitic Infection in the Liver

Below, we discuss the recent understanding of the relationship between miRNAs and various parasitic diseases in humans, including schistosomiasis, clonorchiasis, echinococcosis, and fascioliasis.

### 5.1. Schistosomiasis

Schistosomiasis (also known as bilharzia) is a parasitic infectious disease caused by blood flukes of the *Schistosoma* genus and is one of the most prevalent zoonotic diseases worldwide [110]. *Schistosoma mansoni* (mainly found in Africa, South America, the Caribbean, and the Middle East), *Schistosoma haematobium* (Africa and the Middle East), and *Schistosoma japonicum* (China and southeast Asia) are the main causes of this human disease [111]. Schistosomes get into the body from human skin, and schistosome cercariae migrate to the liver via portal-mesenteric vein system. The female worm lays eggs in this system [110]. Recent studies have shown that certain miRNAs regulate the pathogenesis caused by schistosomiasis infection.

In the murine liver, some miRNAs dysregulated during the mid-phase of infection, including mmu-miR-146b and mmu-miR-155, may be involved in the modulation of hepatic inflammation. Enhanced expressions of mmu-miR-146b and mmu-miR-155 may induce the recruitment of B and T lymphocytes in response to antigens secreted by eggs. In addition, mmu-miR-223, mmu-miR-146a/b, mmu-miR-155, mmu-miR-34c, mmu-miR-199, and mmu-miR-134 exhibit peak expression levels during the late phase of infection and may symbolize the development of schistosomal hepatopathy [112]. The roles of miRNAs and the pathogenesis of hepatic fibrosis in schistosomiasis by both *S. japonicum* and *S. mansoni* were recently reported, which highlighted their roles in regulating antifibrotic and profibrotic mechanisms [111]. It was shown that miR-150-5p, miR-146b-5p, miR-143-3p, miR-199a-3p, miR-10a-5p, miR-4521, miR-31-5p, miR-222-3p, and miR-221-3p were upregulated, whereas miR-663b was downregulated. Furthermore, the authors described that the predicted target genes of the abovementioned miRNAs, including KN motif and ankyrin repeat domains 4, dopamine receptor D1, metallothionein-1H, PL1N1, vanin 1, catenin alpha-3, solute carrier family 39 member, and guanylate-binding protein 5, participated in critical steps related to the progression of hepatic fibrosis, including metabolism, organization of the extracellular matrix proteins, lipid mobilization, and limitation of oxidative stress damage [68]. Schistosomiasis has traditionally been found by detecting eggs in the stool or urine. However, the sensitivities of these examination methods are limited, particularly in patients with a small amount of worm. The sensitivity offered by serologic tests is greater, but their outcomes remain positive in spite of treatment and, therefore, cannot be applied for patient follow-up. MicroRNAs appear to be effective in solving these problems. Recently, characterizing circulating miRNAs secreted by *Schistosoma* spp. have brought about the new possibility of parasite-derived miRNAs as potential biomarkers for the detection of schistosomiasis. Circulating parasite-specific miRNAs, including sja-miR-277 and sja-miR-3479-3p, have demonstrated the possibility of being powerful biomarkers for the diagnosis of *S. japonicum* [113] and *S. mansoni* [114]. In addition, several schistosomal miRNAs, including bantam, miR-2c-3p, miR-3488, and miR-2a-5p, were isolated from extracellular vesicles from the sera of *Schistosoma*-infected individuals. This suggests that these miRNAs can be applied both for diagnostic and monitoring markers [67].

### 5.2. Clonorchiasis

Clonorchiasis, which is one of the most common zoonoses and a prevalent food-borne parasitic disease, is caused by *Clonorchis sinensis*. Over 200 million people are estimated to be at risk for *C. sinensis* infection, and over 15 million people have *C. sinensis* infections worldwide [115]. Persistent infections of *C. sinensis* give rise to the progression of hepatobiliary diseases, including cholangitis, cholelithiasis, cholecystitis, pancreatitis, hepatic fibrosis, cholangiocarcinoma, and liver cancer [116]. Although the molecular mechanisms of carcinogenesis associated with clonorchiasis are not fully understood, some studies have linked changes in miRNA expression patterns to specific biological reactions and carcinogenesis caused by clonorchiasis.

MicroR-71 and its family members comprised the highest proportion of total reads in a study investigating the role of miRNAs in parasitic infections, and the miR-71 family was shown to be preserved in *S. mansoni*, *S. japonicum*, *Ixodes scapularis*, and *Anopheles gambiae*, indicating that miR-71 has a pivotal role in flatworms [117]. Using a rat model of clonorchiasis, miRNAs with different expression profiles were detected, and their roles were also mentioned. The main function of miR-335, which was reduced by almost 6-fold, is to inhibit the proliferation and migration of hepatic stellate cells [118,119]. In vitro experiments showed that the expression of let-7i, a tumor suppressor miRNA, which was associated with the excretory-secretory protein (ESP)-induced TLR4 upregulation and contributed to host immune responses against liver fluke infection, was decreased in human HuCCT1 cholangiocarcinoma cells treated with *C. sinensis* ESP. Real-time quantitative polymerase chain reaction analysis using ESP-treated normal cholangiocytes (H69 cells) revealed that the expression of nine miRNAs (miR-16-2, miR-93, miR-95, miR-153, miR-195, miR-199-3P, let7a, let7i, and miR-124a) were similarly modulated, indicating that the regulation of cell growth by these miRNAs is common to both cancerous and non-cancerous cells [66]. Thus, identifying key targets and miRNA-related genes associated with multiple oncogenic pathways during *C. sinensis* infection is important for determining the disease diagnosis and prognosis.

### 5.3. Echinococcosis

Human cystic and alveolar echinococcoses are zoonoses caused by infections of larval-stage *Echinococcus granulosus* and *Echinococcus multilocularis* [120]. Cystic echinococcosis (CE) is categorized as a major public health concern [121]. Alveolar echinococcosis (AE) is one of the most hazardous human parasitic zoonoses. This echinococcal metacestode grows in the liver and creates an alveolar-like structure, which is composed of several vesicles surrounded by a large granuloma. Human AE is a severe and emerging disease that, with delayed diagnosis, can be fatal [122].

In liver tissue from patients with CE, miR-19 expression was significantly diminished compared with that in normal liver samples. *E. granulosus* can suppress miR-19 expression and induce fibrosis via an increase in TβRII expression and activation of hepatic stellate cells [69]. In addition, egr-miR-71 and egr-let-7 can be detected in human serum during hydatid cyst infection; thus, they can be applied as potential biomarkers for the early diagnosis and monitoring of CE [121]. MicroR-483-3p is a potential circulating marker for AE, which targets the lamin-B receptor and has been involved in cancer development. MicroR-483-3p is upregulated in the serum of patients with AD as compared with normal controls, which provides a new methodology for the development of a diagnostic biomarker of AE [70].

### 5.4. Fascioliasis

Fasciolosis, which is one of the most widespread helminthic diseases, is distributed worldwide, especially in the Americas, Europe, South Africa, the Middle East, and Asia [123,124,125]. According to conservative estimates, 2.4–17 million people are infected with *Fasciola hepatica*. An additional 180 million people worldwide are at a risk for infection.

Four parasite-specific miRNAs were identified in the serum of *Fasciola gigantica*-infected buffaloes, and fgi-miR-87 and fgi-miR-71 were specifically detected. Circulating emu-miR-71 can regulate nitric oxide production by macrophages, suggesting that circulating parasite-derived miRNAs play roles in host–parasite interactions [126]. Studies have shown that miR-87 may be involved in anti-pathogen immune responses [127]. MicroRNAs were identified in the extracellular vesicles (EVs) of the trematode species *Dicrocoelium dendriticum* and *F. hepatica*, and potential immune-regulatory miRNAs and host targets were identified. The two members of eumetazoan miR-10 family, which are the most highly enriched miRNAs in EVs of both species, have eight orthologs in the host *F. hepatica* (bta-mir-10a, bta-mir-10b, bta-mir-99a, bta-mir-99b, bta-mir-100, bta-mir-125a, bta-mir-125-1, and bta-mir-125-2b). Previously known miR-125 members of the human miR-10 family were shown to play critical roles in the development of the immune system, immunological host defense, and cancer [128]. In addition, enrichment of let-7 family members and miR-277 in EV-derived datasets was speculated to be associated with immunomodulatory effects in murine hosts of nematodes [129].

## 6. Conclusions

Some liver miRNAs are beneficial for refractory infectious liver diseases. Accumulating evidence has revealed that miRNAs play important roles in many biological processes involved in liver infectious diseases, including viral hepatitis, liver abscesses due to bacterial infections, and parasitic infections. Many studies have been conducted to investigate the immunological interactions of various pathogens and their relationships with miRNA-expression levels (including those of circulating miRNAs) as potential biomarkers of infectious liver diseases. Moreover, elucidating the mechanistic details of the immune response and its regulatory miRNAs is likely to be beneficial for developing immune therapies. Interestingly, miRNAs are not immunogenic because they do not encode proteins. A combination between conventional and miRNA-based therapies may be beneficial for treating refractory infectious liver diseases. Several problems need to be resolved before miRNAs can be applied in clinical practice. First, various autoimmune diseases may occur if miRNAs enhance uncontrolled immune reactions. Second, miRNA modulation in vivo is still unpredictable since the regulation of miRNA in vivo may not always be observed due to inherent complexities. Therefore, extensive in vivo confirmation of miRNA regulation is required. Finally, global miRNA research associated with immunopathology will elucidate the utility of clinical miRNA applications against infectious liver diseases.

## Figures and Tables

**Table 1:** Altered miRNA expressions in various infectious liver diseases.

	Pathogen	miRNA	Expression	Target Molecules or Signaling Pathways	Reference
Hepatitis B Virus	HBV	let-7	down	TLR4 and STAT3 signaling	[40]
	HBV	miR-21	up	STAT3 signaling	[41,42]
	HBV	miR-122	down	SOCS3	[43]
	HBV	miR-372, miR-373	up	NFIB-dependent signaling	[44]
Hepatitis C Virus	HCV	miR-21	up	TLR, IRAK1 and 4, TRAF6, MyD88 signaling	[45]
	HCV	miR-122	up	SOCS3, STAT3, ISGF3	[46,47]
	HCV	miR-125b	down	TLR2/MyD88 signaling	[48]
	HCV	miR-130a	up	IFITM1, IFNα/IFNβ, ISG15, USP18, MxA	[49,50]
	HCV	miR-146a	up and down	Unknown	[51]
	HCV	miR-155	up	IFN-related molecules	[52,53,54]
	HCV	miR-196a	up	Bach1, HMOX1	[55]
	HCV	miR-373	up	IRF9, JAK1, JAK/STAT signaling	[56]
Bacteria	*C. trachomatis*	miR-9	up	Unknown	[57,58]
	*C. trachomatis*	miR-15a	down	Unknown	[57,58]
	*C. trachomatis*	miR-16	down	Unknown	[57,58]
	*C. trachomatis*	miR-19	up	Unknown	[57,58]
	*C. trachomatis*	miR-29	down	Unknown	[57,58]
	*Enterobacteriaceae* spp.	miR-122	down	Unknown	[59]
	*Mycobacterium tuberculosis* (*Hepatic*)	miR-149 (rs2292832)	down	Unknown	[60]
	*Enterobacteriaceae* spp.	miR-155	up	Unknown	[61,62]
	*C. trachomatis*	miR-182	down	Unknown	[63]
	*C. trachomatis*	miR-200	down	Unknown	[57,58]
	*C. trachomatis*	miR-203	down	Unknown	[57,58]
	*C. trachomatis*	miR-204	down	Unknown	[57,58]
	*C. trachomatis*	miR-214	down	ICAM1	[64]
	*Enterobacteriaceae* spp.	miR-223	up	Unknown	[65]
	*C. trachomatis*	miR-429	down	Unknown	[57,58]
	*C. trachomatis*	miR-451	up	Unknown	[57,58]
Parasite	*C. sinensis*	let-7a	up	Unknown	[66]
	*C. sinensis*	let-7i	up	TLR4 signaling	[66]
	*S. japonicum* and *S. mansoni*	miR-2a-5p	up	Unknown	[67]
	*S. japonicum* and *S. mansoni*	miR-2c-3p	up	Unknown	[67]
	*S. japonicum* and *S. mansoni*	miR-10a-5p	up	Unknown	[68]
	*C. sinensis*	miR-16-2	up	Unknown	[66]
	*E. granulosus*	miR-19	down	TβRII	[69]
	*S. japonicum* and *S. mansoni*	miR-31-5p	up	Unknown	[68]
	*C. sinensis*	miR-71	up	Unknown	[69]
	*C. sinensis*	miR-93	up	Unknown	[66]
	*C. sinensis*	miR-95	up	Unknown	[66]
	*C. sinensis*	miR-124a	up	Unknown	[66]
	*S. japonicum* and *S. mansoni*	miR-143-3p	up	Unknown	[68]
	*S. japonicum* and *S. mansoni*	miR-146b-5p	up	Unknown	[68]
	*S. japonicum* and *S. mansoni*	miR-150-5p	up	Unknown	[68]
	*C. sinensis*	miR-153	up	Unknown	[66]
	*C. sinensis*	miR-195	up	Unknown	[66]
	*S. japonicum* and *S. mansoni*	miR-199a-3p	up	Unknown	[66,68]
	*S. japonicum* and *S. mansoni*	miR-221-3p	up	Unknown	[68]
	*S. japonicum* and *S. mansoni*	miR-222-3p	up	Unknown	[68]
	*C. sinensis*	miR-355	down	Unknown	[69]
	*E. granulosus*	miR-483-3p	up	LBR	[70]
	*S. japonicum* and *S. mansoni*	miR-663b	down	Unknown	[68]
	*S. japonicum* and *S. mansoni*	miR-3488	up	Unknown	[67]
	*S. japonicum* and *S. mansoni*	miR-4521	up	Unknown	[68]
